# Haemoglobinuria among children with severe malaria attending tertiary care in Ibadan, Nigeria

**DOI:** 10.1186/1475-2875-11-336

**Published:** 2012-10-05

**Authors:** Wasiu A Ajetunmobi, Adebola E Orimadegun, Biobele J Brown, Nathaniel K Afolabi, Folorunso A Olabiyi, John I Anetor, Samuel Omokhodion, Kikelomo Osinusi, Felix O Akinbami, Wuraola A Shokunbi, Olugbemiro Sodeinde, Delmiro Fernandez-Reyes

**Affiliations:** 1 Department of Paediatrics, College of Medicine, University of Ibadan, University College Hospital, Ibadan, Nigeria; 2 Department of Haematology, College of Medicine, University of Ibadan, University College Hospital, Ibadan, Nigeria; 3 Childhood Malaria Research Group, University College Hospital, Ibadan, Nigeria; 4 Department of Chemical Pathology, College of Medicine, University of Ibadan, University College Hospital, Ibadan, Nigeria; 5 Division of Parasitology, Medical Research Council National Institute for Medical Research, London, UK

**Keywords:** Childhood severe malaria, Haemoglobinuria

## Abstract

**Background:**

Haemoglobinuria is one of the manifestations of severe malaria and results from severe intravascular haemolysis. Glucose-6-phosphate dehydrogenase (G6PD) deficiency has been implicated in its aetiology. Haemoglobinuria may be associated with severe anaemia and, less frequently, acute renal failure.

**Methods:**

A prospective case-control study was carried out to determine the incidence of haemoglobinuria as confirmed by dipstick urinalysis, microscopy and spectrophotometric measurement, among children with severe malaria. A total of 251 children presenting at the Children’s Emergency Ward with severe malaria were recruited over a period of 21 months. The G6PD status and the outcomes of severe malaria in children with and without haemoglobinuria was studied with respect to renal failure, the recurrence of haemoglobinuria and blood pressure changes over a three-month follow-up period.

**Results:**

It was found that the incidence of haemoglobinuria among children with severe malaria is 19.1%. Children <5 years constituted 76.8% of all the study patients. Patients with haemoglobinuria had median age of 52.5 months, which was significantly higher than 35 months in patients without haemoglobinuria (p=0.001). Although, haemaglobinuria was commoner among boys (54.2%) than girls (45.8%), the difference was not statistically significant. There were no significant differences between children with and without haemoglobinuria regarding their nutritional status or parasite densities. Among the clinical features of the study patients, only jaundice was significantly associated with haemoglobinuria (p=0.0001). Renal failure occurred in three out of 48 children with haemoglobinuria and in none of the 203 without. There was not recurrence of haemoglobinuria in the follow-up period. At discharge, blood pressure was elevated in six children (one previously haemoglobinuric), but all returned to normal within the follow-up period.

**Conclusions:**

Haemoglobinuria was a prominent feature of severe malaria and it was significantly associated with jaundice at presentation. Haemoglobinuria was commoner in older children than younger children but not related to sex. G6PD deficiency was not an independent predictor of the occurrence or outcome of haemoglobinuria. Blood pressure was not affected by haemoglobinuria on admission nor during follow-up.

## Background

It is estimated that 300 million clinical cases of malaria occur each year in malaria-endemic regions. Most of the deaths from malaria are due to the severe forms of the disease, especially cerebral malaria and severe malarial anaemia [1]. Recent reports point to a reduction of malaria cases in parts of Africa [2]. However, Nigeria, the most populous country of Africa, accounts for a quarter of the global cases and a third of the malaria-attributable childhood deaths [1,3-5]. In Nigeria, *Plasmodium falciparum* accounts for 96-98% of malaria infections, while other infections are due to *Plasmodium malariae*[6,7].

Malarial illnesses may take a variety of clinical forms, differing in pattern and severity, from uncomplicated to severe and complicated malaria. Severe *falciparum* malaria may manifest with wide variety of features as described by WHO haemoglobinuria being one of them [8]. Haemoglobinuria occurs when the capacity of haptoglobin to bind free haemoglobin is exceeded. Despite their sometimes similar visual appearances, the absence of red blood cells on microscopic examination distinguishes haemoglobinuria from haematuria.

Retrospective studies of malaria have reported haemoglobinuria incidence in adults ranging from 15% to 63% [9,10]. Some other studies have documented haemoglobinuria as rare in children [11,12] while other studies have reported incidence rates ranging from 2% to 17% [13-16] across different malaria-endemic regions. A multicentre study [17] of severe malaria among children in 10 African countries, Nigeria inclusive, reported a mean haemoglobinuria prevalence of 3.3%. Similarly, a prospective study of 627 children with confirmed diagnosis of malaria in Maiduguri, Nigeria, found haemoglobinuria in 3.7% of the patients [18]. A common problem of these studies is that they do not indicate the number of severe malaria patients included in the analyses.

Several factors such as immune hypersensitivity in long-term residents of *P. falciparum*-endemic areas who took quinine irregularly or glucose-6-phosphate dehydrogenase (G6PD) deficiency have been implicated in the aetiology of haemoglobinuria. Haemoglobinuria can also occur in patients with normal erythrocyte G6PD levels who receive quinine for severe malaria [19,20]. However, there is lack of current data from prospective studies regarding the correlation between haemoglobinuria and severe malaria, with or without G6PD deficiency, in Nigerian children [21].

The clinical features peculiar to malaria-induced haemoglobinuria are mainly features of intravascular haemolysis and these features may be associated with anaemia. They generally include fever, abdominal pain, back pain or pain in the limbs, dyspnoea, tachycardia, a non-specific complaint of generalized malaise or dizziness [7,22]. Shock and renal failure can occur. However, in some cases, patients can be asymptomatic, their pallor being only detected by family members or other individuals. The most consistent findings in malaria haemoglobinuria are fever, pallor, jaundice and passage of dark urine [8].

Haemoglobinuria was previously thought to be innocuous and self-limiting, usually resolving within three days of onset. However, studies have shown that haemogobinuria may result in acute renal failure. Krishna and Karnad [10] in a prospective study of 301 severe malaria patients in India reported that haemogobinuria accounted or 33% of the mortality.

The prevalence of haemoglobinuria, its clinical profile, the associated frequency of G6PD deficiency and its effects on the outcome of severe malaria among Nigerian children, are unknown. In the present study, haemoglobinuria among children with severe malaria was prospectively investigated using a quantitative spectrophotometric method [23] as distinct from qualitative spectroscopic detection. The incidence of haemoglobinuria, its association with clinical features of malaria and the G6PD status was documented among children with severe malaria attending the tertiary care facility at the University College Hospital, Ibadan.

## Methods

### Study site

All study participants were recruited under the auspices of the Childhood Malaria Research Group (CMRG) at the 600-bed tertiary hospital, University College Hospital (UCH) in the city of Ibadan, Nigeria in west sub-Saharan Africa. Ibadan is the capital city of Oyo State in the South-West of Nigeria. It has a population of 2,550,293 people and annual growth rate of 2.83% (2006 census) [24]. The population of Ibadan comprises predominantly the Yoruba-speaking people of the western Nigeria.

This study was carried out at the Otunba Tunwase Children Emergency Ward (OTCHEW) of the Department of Paediatrics of the University College Hospital, Ibadan. The OTCHEW is one of the six wards in the Department of Paediatrics and it has thirty-five bed-spaces for admission. The number of admissions per year is approximately 2,000. About twice that number are treated there without needing admission.

### Study design and case definitions

This was a prospective case control study. After obtaining informed consent, children were recruited between the ages of six months and twelve years using WHO severe malaria criteria [8]. Cerebral malaria (CM) cases were defined as children in unrousable coma for at least one hour in the presence of asexual *P. falciparum* parasitaemia with normal cerebrospinal fluid. A Blantyre coma score less or equal to 2 was used to define coma status. Severe malarial anemia (SMA) cases were defined as conscious children with packed cell volume (PCV) less than 16% in the presence of *P. falciparum* parasitaemia. Other forms of severe malaria (OFSM) were defined according to WHO criteria [8] and required hospitalization. These included children with prostration, haemoglobinuria, two or more seizures within 24 h or hyperpyrexia. Renal failure was defined as urine output <12 ml/kg/24 h or a plasma creatinine concentration above the age-related normal range, persisting after rehydration [8].

The cases were defined as children with severe malaria presenting with haemoglobinuria. Age- and sex-matched severe malaria patients without haemoglbinuria served as controls. Age matching criteria were +/− three months for six to 24 months and +/− six months for older children. Children were excluded from the study if they had been transfused within three months before presentation, or there was history of severe trauma or burns within the preceding month or they had a haemoglobinopathy or they presented with haematuria.

Peripheral blood films, thick and thin, for microscopic examination for malaria parasites were carried out on admission. About 5ml of venous blood was withdrawn from each study participant into EDTA tubes for haemoglobin electrophoresis and G6PD screening. The blood pressure of each patient presenting with severe malaria was measured on admission.

Dipstick urinalysis was carried out in all enrolled children. Urine samples that tested positive for blood were divided into two aliquots. Urine microscopy was carried out on one aliquot to exclude haematuria while the second was preserved at −20°C to measure haemoblogin concentration. Urine racks containing aliquots from each time the child passed urine, were kept for all patients with dark coloured urine to monitor the clearing of the urine.

Children with haemoglobinuria and their matched controls were followed up during the 2^nd^, 4^th^, 8^th^ and 12^th^ weeks after recruitment. At each follow-up clinic visit, children were screened for malaria parasites and their clinical status, including blood pressure measurements, was documented. Urinalysis was repeated for both the cases and the controls. Those who tested positive for blood had urine microscopy repeated to exclude haematuria.

### Urine collection procedure and urinalysis

About 10ml of urine was collected and subjected to urinalysis immediately. Otherwise, it was stored in the fridge at 4°C and the urinalysis done within 24 h. Multistix strip (DIALAB Ref G04010C) [25] was completely immersed in each of the urine samples, in clean universal bottles, after gently but thoroughly shaking the sample. Samples testing positive for blood were divided into two. Half of the sample was immediately sent to the microbiology laboratory for urine microscopy. The other half was preserved at −20°C for spectrophotometry in batches. Haemoglobinuria was assessed using the rate spectrophotometry method described by Chalmer and Snell [23].

### Data collection procedure

Each child’s caregiver was interviewed using a structured project questionnaire. History was obtained from the caregiver and, if old enough, the child. A complete physical examination was carried out on each patient. All findings from physical examination and other relevant information including the results of G6PD test, PCV, parasite counts, haemoglobin electrophoresis were recorded into the structured project questionnaire.

### Packed cell volume

PCV was determined using the capillary method and spinning in a Hawksley micro-haematocrit centrifuge at 11,000g for 5 min at room temperature. The packed cell volume was then read with a haematocrit reader.

### Malaria parasite screening

Thick and thin films were made on the same slide for each patient. The thin film was fixed with methanol immediately and the slide was allowed to dry, then flooded with Giemsa already diluted 1 in 10 with phosphate buffer, pH 7.2. The stain was washed off with distilled water, after allowing it to stand for about 10 min, and then air-dried. The stained films were then examined microscopically for malaria parasites using a x100 objective (oil immersion) and x10 eye piece lenses i.e. total magnification of x1,000. The parasites were counted against 200 white blood cells (WBC) and parasite density was calculated for each patient based on an assumed total WBC of 8000/μL of blood.

### Determination of G6PD status by fluorescent spot test

G6PD status of recruited children was determined using the fluorescent spot test method of Beutler and Mitchell [26].

### Data management and analysis

Data was entered into a microcomputer and analyzed using the Epi-Info Version 6 and SPSS 11.0 for windows. Categorical variables were compared using either the uncorrected chi-square test or Fisher’s exact test while continuous variables were analyzed using the Student’s *t-*test. Data not normally distributed were compared using the Mann-Whitney *U*-test. Continuous variable estimates were expressed as mean (±SD) or median (inter-quartile range) while categorical variables were in proportions, ratios and percentages. Statistical significance level was set at p<0.05.

## Results

### Characteristics of study patients

A total of 265 children with severe malaria were screened over a period of 21 months (January 2006 to September 2007) and 251 eligible children were recruited into this study. These comprise 102 (40.6%) females and 149 (59.4%) males giving a female to male ratio of 1:1.5 (Table 1). The remaining 14 patients were excluded due to incomplete data. The 265 severe malaria patients represented 3.05% of the 8,705 children with malaria-positive smears seen over the study period.

**Table 1:** Characteristics of study patients

	**Haemoglobinuria**		**All**
	**Positive**	**Negative**	
**Number of Patients N (%)**	48 (19.1)	203 (80.9)	251
**Sex Ratio** Female : Male	22 : 26	80 : 123	102 : 149
**Age Months** Median (IQR)	52.5 (18–138)	35 (7–137)*	36 (7–138)
**Age Distribution N (%)**			
6–11 months	0 (0)	18 (8.9)	18 (7.2)
12–23 months	4 (8.3)	42 (20.7)	46 (18.3)
24–35 months	12 (25)	43 (21.2)	55 (21.9)
36–47 months	6 (12.5)	33 (16.3)	39 (15.5)
48–59 months	8 (16.7)	27 (13.3)	35 (13.9)
> 59 months	18 (37.5)	40 (19.7)	58 (23.2)
**Weight for Age [z-score]** Median (IQR)	−1.32 (−5.38 - 1.45)	−1.31 (−6.22 - 4.06)	−1.31 (−6.22 - 4.06)
**Parasite Density** Geometric Mean Range	8960.9	9209.6	9161.5
	607–198,606	64–192,000	64-198606
**G6PD Status** Deficient : Normal N(%)			
Males	4 : 22 (15 : 85)	15 : 108 (12 : 88)	19 : 130 (13 : 87)
Females	0 : 22 (0 : 100)	4 : 76 (5 : 95)	4 : 98 (4 : 96)
**Clinical forms of Severe Malaria** N (%)			
Cerebral Malaria	17 (35.4)	70 (34.5)	87 (34.7)
Severe Malarial Anaemia	13 (27.1)	90 (44.3)	103 (41)
Other Forms of Severe Malaria**	18 (37.5)	43 (21.w)	61 (24.3)
**Outcome** N (%)			
Survived	42 (83.5)	189 (93.1)	231 (92)
Died	6 (12.5)	14 (6.9)	20 (8)

* p significant at < 0.05.

** Postration (82%); Haemaglobinuria (8%); Seizures >2 episodes/day (5%); Hyperpyrexia (5%).

The characteristics of the studied patients are summarized in Table 1. Forty-eight (19.1%) of the patients had haemoglobinuria. These consisted of 22 (45.8%) females and 26 (54.2%) males. Overall, the ages of the patients ranged between six months and 12 years with the median age being 36 months. The median age of the patients with haemoglobinuria was 52.5 months, significantly higher than 35 months in patients without haemoglobinuria (p = 0.001). Infants aged six to 11 months constituted 7.2% of the study patients. Children under five years of age (6 to 59 months) constituted 76.8% of all the study patients. Children over five years of age constituted 23.2% of all patients (Table 1). A significantly higher proportion (37.5%) of patients with haemogobinuria were five years and older, compared to (19.7%) in patients without haemoglobinuria (Table 1). There was no statistically significant difference in the age distribution of patients by gender. The patients’ weights ranged from 5.6kg to 72.5kg with an overall mean weight of 13.74±6.3kg. The mean weight for the patients with haemoglobinuria was 15.29±5.5kg compared with 13.38±6.4kg in those without haemoglobinuria. The mean weight–for-age Z-score of all patients was −1.36. The median weight for age Z-scores were −1.32 and −1.31 among the patients with haemoglobinuria and patients without haemoglobinuria, respectively (Table 1). There was no statistically significant difference in the nutritional status of patients with and without haemogobinuria. The range and the geometric mean parasite counts of patients with haemoglobinuria were slightly lower than those without haemoglobinuria, but these were not statistically significant (Table 1). There was no significant difference in the G6PD status of patients with or without haemoglobinuria even after stratifying according to gender. However, it is noteworthy that all the G6PD deficient patients who had haemoglobinuria were males (Table 1).

### Clinical forms of severe malaria

The distributions of patients by clinical forms of severe malaria are shown in Table 1. Generally, CM, SMA and OFSM accounted for 34.7%, 41% and 24.3% of the study patients respectively. Among those with haemoglobinuria, CM, SMA and OFSM accounted for 35.4%, 27.1% and 37.5% respectively. All the patients with CM were treated with intravenous quinine (according to the Nigerian national policy at the time) and none developed haemoglobinuria during treatment. Those who presented with haemoglobinuria did not have their condition worsened by quinine treatment.

The overall mortality in this study was 20 (8%). These consisted of 6 (12.5%) of the 48 patients with haemoglobinuria compared with 14 (6.9%) of the 203 patients without haemoglobinuria (Table 1). Haemoglobinuria was present in 30% of the patients who died. The difference in mortality between patients with versus without haemoglobinuria was not statistically significant. One of the dead patients with haemoglobinuria had anuric acute renal failure, while the other two patients who developed renal failure secondary to malarial haemoglobinuria survived after dialysis. This showed that acute renal failure occurred in 6.25% of the patients with haemoglobinuria and 1.2% of all the severe malaria patients. None of the patients without haemoglobinuria had acute renal failure. The distribution of the mortality among CM, SMA and OFSM was 11 (55%), five (25%) and four (20%) of the total mortality respectively.

### Clinical features

The comparisons of the clinical features of malaria in patients with and without haemoglobinuria are shown in Table 2. The proportion of patients who presented with vomiting, pallor, passage of dark urine or reduced urine frequency was higher in patients with haemoglobinuria than patients without haemoglobinuria. However, a statistically significance difference was only seen with jaundice and passage of dark urine (Table 2). The proportions of other symptoms and signs were similar between the two groups.

**Table 2:** Symptoms and signs of study patients

	**Haemoglobinuria**		**OR**	**95% CI**
	**Positive**	**Negative**		
**Presenting Complains** N (%)				
Vomiting	36 (75)	127 (62.6)	1.8	0.83 - 3.93
Pallor	36 (75)	139 (68.5)	1.38	0.64 - 3.04
Yellowness of the eyes	21 (43.8)	44 (21.7)	2.81*	1.37 - 5.77
Difficulty with breathing	13 (27.1)	73 (36)	0.66	0.31 - 1.41
Passage of dark urine	35 (72.9)	49 (24.1)	8.46*	3.91 - 18.56
Convulsion	22 (45.8)	109 (53.7)	0.77	0.38 - 1.37
Body swelling	2 (4.2)	8 (3.9)	1.06	0–5.73
Reduced frequency of urination	4 (8.3)	13 (6.4)	1.33	0.34 - 4.72
**Clinical Signs** N (%)				
Pallor	39 (81.3)	160 (78.8)	1.16	0.49 - 2.83
Temperature > 37.4 C	40 (83.3)	166 (81.8)	1.11	0.48 - 2.57
Liver size > 2cm	32 (66.7)	127 (62.6)	1.19	0.61 - 2.32
Splenomegaly	10 (20.8)	66 (32.5)	0.54	0.25 - 1.16
Jaundice	28 (58.3)	55 (27.1)	3.77*	1.86 - 7.67
Respiratory distress	14 (29.2)	71 (35)	0.76	0.38 - 1.52

* p significant at < 0.05.

On admission, 48 of the 251 severe malaria patients had their urine testing positive for haemoglobin and had haematuria excluded by urine microscopy. The haemoglobin concentrations of urine samples of 45 of the patients with haemoglobinuria ranged from 5 to 6,800mg/l (median 400mg/l). Three samples were lost during storage.

### Follow-up

Out of the 42 patients with haemoglobinuria who survived 19 (45%), 29 (69%), 13 (31%) and 27 (64%) were followed up at two weeks, four weeks, eight weeks and 12 weeks respectively. For those without haemoglobinuria 24 (12.6%), 39 (20.6%), nine (4.7%) and 44 (23.2%) were followed up at 2, 4, 8 and 12 weeks respectively. The follow-up at three months involved 15 males (55.6%) and 12 (44.4%) females with haemoglobinuria and 30 (68.2%) males and 14 (31.8%) without haemoglobinuria. Two of the patients with haemoglobinuria had recurrent haemoglobinuria at one month while haemoglobinuria never occurred in the group of patients without initial haemoglobinuria.

The blood pressures of the study patients on admission and during follow-up are shown in Table 3. There was no significant difference in the mean systolic or diastolic blood pressures of patients with or without haemoglobinuria on admission and follow-up at one month and three months (Table 3). Only 6 (2.3%) of the study patients had transient elevated blood pressure on admission. These comprised 1 (2.1%) of the patients with haemoglobinuria and 5 (2.5%) of the patients without haemoglobinuria. Subsequently, the blood pressures of all the studied patients were normal.

**Table 3:** Blood Pressures of Study Participants

	**Haemoglobinuria**	
	**Positive**	**Negative**
**BP at Admission** mean (sd)		
Systolic	94.3 (10.8)	92.1 (10.1)
Diastolic	55.4 (6.8)	55.2 (8.2)
**BP at 1 Month** mean (sd)		
Systolic	93.1 (6.5)	91.9 (6.9)
Diastolic	57.6 (6.4)	56.2 (6.3)
**BP at 3 Months** mean (sd)		
Systolic	90 (6.9)	90.3 (7.1)
Diastolic	56 (6.4)	57.6 (5.9)

## Discussion

The present study has shown that the incidence of haemoglobinuria among children with severe malaria is 19.1%. This value is higher than the previously reported range of 2% to 17% [9,14,17,18] but lower than other studies in adults which reported incidence ranging from 63% to 70% [10,13]. The different figures from previous studies could be due to the fact that haemoglobinuria was diagnosed only by visual inspection of the urine samples, or the inclusion of all malaria cases as the denominator. On the contrary, we have focused only on those children with severe malaria. All our haemoglobinuric patients had dark urine at admission. Although we cannot exclude concomitant myoglobinuria, previous studies have reported that dark urine is generally the result of intravascular haemolysis [22].

There was a significant difference in the age-distribution of patients with or without haemoglobinuria. The proportion of children above five years with haemoglobinuria was higher than those without haemoglobinuria. This finding suggests that haemoglobinuria may be commoner in children above five years. A previous prospective study reported a higher incidence of haemoglobinuria among those more than four years old [27]. However, that study only used dipstick to verify diagnosis of haemoglobinuria and therefore it cannot exclude microscopic haematuria. However in the present study a more accurate estimate of haemoglobinuria was obtained by combining urine dipstick testing with microscopy and spectrophotometric methods.

The proportion of boys in our study was higher than girls, a finding that is consistent with previous studies from the same centre [28,29]. Though the proportion of males with haemoglobinuria was higher than females, there was no significant gender predominance among the patients with or without haemoglobinuria. The gender distribution of subjects enrolled for this study reflected the general pattern of admissions into the emergency ward.

There was no significant difference in the nutritional status of patients with or without haemoglobinuria. The weight-for-age z-score was consistent with previous findings that severe malaria generally occurring in children whose nutritional is within normal limits [28,30].

Almost half of the study subjects were SMA patients, similar to what has been reported in studies from Yemen [31], Sudan [32], and Uganda [14].

Jaundice was significantly associated with haemoglobinuria, which is in accordance with previous studies on blackwater fever that have reported jaundice and pallor as major manifestations [7,20,33,34]. Although not statistically significant, a higher proportion of patients with clinical pallor were observed among the haemoglobinuria group.

Occurrence of haemoglobinuria has been previously linked with parasitaemia. While some researchers have associated blackwater fever with scanty or no parasitaemia [7,20], others reported higher incidence rates of haemoglobinuria in areas of high malaria transmission and parasitaemia [27]. However, a previous study in Uganda showed no significant difference in the prevalence of haemoglobinuria in areas with low, moderate or high parasite transmission [16]. The present study did not show any significant difference in the parasite density of patients with or without haemoglobinuria in this urban area of high malaria transmission. It is, therefore, not likely that the parasite density alone could explain the high frequency of haemoglobinuria found in the present study.

The frequency of G6PD deficiency among severe malaria patients in the present study was 9.2%. This was close to the frequency of 6% reported from the same centre in the 1960’s [35]. The frequency of G6PD deficiency was higher among boys compared to girls. This is consistent with the distribution pattern of G6PD deficiency in this population. G6PD deficiency being a sex-linked disorder occurs more commonly in the male population. The G6PD deficiency frequency was significantly lower in our hospital population than the one obtained from a previous study of a healthy homogenous population in the community [36]. Virtually by definition, hospital populations are already selected by their help-seeking behaviour due to illness (malaria in this case). In the present study, they have further been selected by the admission criteria. Thus, the observed reduced frequency of G6PD-deficiency is consistent with the hypothesis that G6PD-deficiency protects against severe malaria. There was no significant difference in the G6PD status of patients with or without haemoglobinuria even after stratifying according to gender. The lack of difference in the occurrence of haemoglobinuria between G6PD-normal and deficient children suggests that G6PD deficiency is not a significant independent determinant in patients with severe malaria.

Previous studies have implicated the repeated exposure to quinine in the aetiology of Blackwater fever [7,20,34]. We cannot comment on the role of previously administered quinine in the pre-hospital appearance of haemoglobinuria. However, none of the CM patients who presented without haemoglobinuria and were treated with quinine, developed haemoglobinuria. In addition, haemoglobinuria was not worsened among the CM patients who had haemoglobinuria on admission and were treated with quinine. In any case, since the completion of this study, in the revised National Malaria Policy, Quinine is no longer recommended for routine use as a first-line drug in the treatment of CM. Thus, the risk of repeated exposure to quinine is minimal.

The overall mortality rate of severe malaria from this study was 8% with CM accounting for majority of the cases; this finding is consistent with previous studies [17]. Haemoglobinuria was present in 30% of the dead patients. One of the dead patients with haemoglobinuria had anuric acute renal failure while the other two patients who developed renal failure secondary to malarial haemoglobinuria survived after dialysis. Acute renal failure occurred in 6.25% of the patients with haemoglobinuria and 1.2% of all the severe malaria patients. The inference that can be drawn from the above findings is that haemoglobinuria is not as innocuous as earlier thought and that malaria- induced acute renal failure should be sought and treated promptly since, 1 (33.3%) of the patients that developed renal failure died. Moreover, haemoglobinuria should be handled with fervent seriousness because all the three patients that had acute renal failure had haemoglobinuria.

The urine of all the patients with haemoglobinuria became visually clear over a period of three days. Comparison of the blood pressure of patients with or without haemoglobinuria showed no significant difference on admission and during follow-up. Even the three patients who had renal failure had normal blood pressure on admission. Of these, the two survivors had normal blood pressure throughout the follow-up period. In all six children (one in the group with haemoglobinuria) who had mild elevation of blood pressure in the acute phase, the blood pressure returned to normal within the follow-up period. This indicates that blood pressure elevation in severe falciparum malaria is transient. However, our period of observation (follow-up) was limited to three months. The possibility of subsequent blood pressure elevation during longer follow-up cannot be excluded. The mechanism of this blood pressure elevation is not clear. The proportions involved are small. But if anything, it is slightly lower in the haemoglobinuric group (1/48 ie 2.1%) than the group without haemoglobinuria (5/203 ie 2.5%). This suggests that direct toxicity of free haemoglobin on renal tissues is not likely to be a major factor. Since *P. falciparum* is known to exhibit cytoadherrence in deep vascular tissues, it is tempting to speculate that the ischaemia consequent upon cytoadherence in the renal vasculature stimulates the rennin-angiotensin mechanism, which raises the blood pressure. The rapid resolution of the rise in blood pressure is consistent with the idea of a rapid reversal of the renal vascular cytoadherence and ischaemia following the clearance of the parasitaemia.

## Competing interests

The authors declare that they have no competing interests.

## Authors’ contributions

DF-R, OS and WAA conceived, designed, deployed and directed the case-controlled study at the Childhood Malaria Research Group, Ibadan, Nigeria. WAA, BJB, AEO, NKA, SO, KO, FOA, WAS, OS and DF-R carried out patient recruitment and follow-up, sample collection, storage and transport. WAA, FAO, JIA carried out haemoglobinuria tests. WAA, BJB, AEO, OS and DF-R analyzed data. DF-R, OS and WAA produced figures and tables and wrote the manuscript. All authors read and approved the final manuscript.
